# Investigation and Characterization of Pickering Emulsion Stabilized by Alkali-Treated Zein (AZ)/Sodium Alginate (SA) Composite Particles

**DOI:** 10.3390/ma16083164

**Published:** 2023-04-17

**Authors:** Ying Kuang, Qinjian Xiao, Yichen Yang, Menglong Liu, Xiaosa Wang, Pengpeng Deng, Kao Wu, Yi Liu, Bo Peng, Fatang Jiang, Cao Li

**Affiliations:** 1 Cooperative Innovation Center of Industrial Fermentation (Ministry of Education & Hubei Province), Hubei Key Laboratory of Industry Microbiology, National “111” Center for Cellular Regulation and Molecular Pharmaceutics, Key Laboratory of Fermentation Engineering (Ministry of Education), Hubei University of Technology, Wuhan 430068, China; lazywawa@163.com (Y.K.); qj_xiao187166@163.com (Q.X.); yangyichen0929123@163.com (Y.Y.); liumenglong0823@163.com (M.L.); wxswyx623@163.com (X.W.); dengpp@whu.edu.cn (P.D.); wukao@mail.hbut.edu.cn (K.W.); liuyi826@hotmail.com (Y.L.); pengbo151040@126.com (B.P.); 2 Department of Architecture and Built Environment, Faculty of Engineering, University of Nottingham, Nottingham NG7 2RD, UK; 3 College of Health Science and Engineering, Hubei University, Wuhan 430062, China

**Keywords:** deamidation, zein, sodium alginate, Pickering emulsion

## Abstract

Pickering emulsions stabilized by food-grade colloidal particles have attracted increasing attention in recent years due to their “surfactant-free” nature. In this study, the alkali-treated zein (AZ) was prepared via restricted alkali deamidation and then combined with sodium alginate (SA) in different ratios to obtain AZ/SA composite particles (ZS), which were used to stabilize Pickering emulsion. The degree of deamidation (DD) and degree of hydrolysis (DH) of AZ were 12.74% and 6.58% respectively, indicating the deamidation occurred mainly in glutamine on the side chain of the protein. After the treatment with alkali, AZ particle size decreased significantly. Moreover, the particle size of ZS with different ratios was all less than 80 nm. when the AZ/SA ratio was 2:1(Z2S1) and 3:1(Z3S1), the three-phase contact angle (*θ*_o/w_) were close to 90°, which was favorable for stabilizing the Pickering emulsion. Furthermore, at a high oil phase fraction (75%), Z3S1-stabilized Pickering emulsions showed the best long-term storage stability within 60 days. Confocal laser scanning microscope (CLSM) observations showed that the water-oil interface was wrapped by a dense layer of Z3S1 particles with non-agglomeration between independent oil droplets. At constant particle concentration, the apparent viscosity of the Pickering emulsions stabilized by Z3S1 gradually decreased with increasing oil phase fraction, and the oil-droplet size and the Turbiscan stability index (TSI) also gradually decreased, exhibiting solid-like behavior. This study provides new ideas for the fabrication of food-grade Pickering emulsions and will extend the future applications of zein-based Pickering emulsions as bioactive ingredient delivery systems.

## 1. Introduction

Pickering emulsions are one of the hot topics in emulsion research in recent years. Compared to traditional emulsions stabilized by surfactants such as Tween and Span, Pickering emulsions are generally stabilized by solid particles and have high stability that is safe, and resistant to agglomeration and Ostwald ripening [1,2]. Traditional inorganic and synthetic nanoparticles, such as SiO_2_ and TiO_2_, are generally less biodegradable and compatible and have certain limitations in their use [3,4]. Contrary to that, natural colloidal particle-stabilized Pickering emulsions have broad development prospects in food and pharmaceutical fields [5,6]. Recently, polysaccharides and proteins such as cellulose, chitosan, starch, zein, whey protein, etc. have been widely studied as Pickering emulsion stabilizers [7,8]. In particular, protein-based particles, have received much attention and have shown high application potential due to their high nutritional density, rich surface groups, and diverse functional characteristics. It has been reported that high-concentration emulsion can improve the adsorption capacity of casein on the emulsion, reduce the oil-water interfacial tension, increase the viscosity of the emulsion system, limit the movement of oil droplets, and increase the stability of the emulsion. In addition, it has also been shown that soy protein has good emulsification properties, which play a significant role in retarding lipolysis. The denaturation of soybean isolate protein at different ionic strengths was investigated and microgel particles were prepared using ultrasonic and high-pressure homogenization methods. Due to its faster adsorption rate at the oil–water interface, O/W emulsion can be better stabilized [7,9,10,11].

Zein is the main component of prolamin extracted from maize with a specific amino acid composition. On the surface of zein particles, there are a lot of hydrophobic amino acids, but there are no charged acidic, basic, or polar amino acids. Due to its hydrophobicity and biocompatibility, zein can be self-assembled into nanoparticles, which can be utilized as oral carriers for the delivery of biologically active hydrophobic substances in the medical and nutritional fields [12,13,14]. However, the strong hydrophobicity also limits its utilization in food and biomedical applications. Recent studies have shown that emulsions or Pickering emulsions prepared from zein are unstable and prone to aggregation and phase separation [15,16]. In addition, changes in pH, ion concentration, temperature, proteases, and other environmental factors are also prone to destabilization, denaturation, and degradation of zein-based carriers [17,18,19,20].

To further improve the stability of the protein-based Pickering emulsion system, biopolymers are usually incorporated to increase their hydrophilicity [21]. It has been reported that the mixture of protein and polysaccharide could significantly improve the hydrophobicity of the protein and improve the stability of the Pickering emulsion [22,23]. Sodium alginate (SA), a linear anionic natural polysaccharide derived from brown algae, is composed of β-d-mannuronic acid (M) and α-l-guluronic acid (G) linked by a 1,4 bond. It is often used to increase the mechanical strength and spatial repulsion of the interfacial layer around the protein-coated oil droplets and enhance the stability of the emulsion [24]. SA has a high content of -COO^−^ and can exhibit polyanionic properties in aqueous solutions. In general, environmental changes affect the ionization and hydrophilicity of -COO^−^ [24,25]. In addition, SA can be adsorbed on the oil–water interface, and the interaction between SA and protein can improve the surface wettability of complex particles, reduce the interfacial tension, and enhance the interfacial activity, which can greatly improve the poor interfacial stability of the zein-stabilized emulsions [26,27].

Therefore, in this study, to improve the interfacial wettability and emulsifying property, zein was first subjected to restricted alkali deamidation, and then alkali-treated zein (AZ) was subsequently obtained via an anti-solvent method. Furthermore, the surface charge density of AZ was adjusted by anionic SA, and non-covalent AZ-SA complexes (ZS) with high negative potential were prepared to stabilize Pickering emulsions with different volume fractions of the oil phase. The oil-droplet size, kinetic stability, interfacial morphology, and rheological properties of the emulsions were also investigated. Moreover, the results show that the addition of anionic SA can significantly increase the charge density on the surface of AZ, promote the irreversible adsorption of the ZS particles at the oil–water interface, and prevent the coalescence or even separation of oil droplets, thus improving the stability of the emulsion system. In addition to investigating the non-covalent interaction between AZ and SA, this work also seeks to understand the stability mechanisms and properties of Pickering emulsions stabilized by ZS particles. In this study, ZS particles were also used as an all-natural, environmentally friendly, safe, and non-toxic emulsifier. On the one hand, it provides a new concept for the preparation of a new green, novel protein-polysaccharide composite food-grade Pickering emulsion, and on the other hand, it has excellent potential for application in the study of fat-substituting foods and human drug delivery. This study aimed to investigate the stabilization mechanism and performance of ZS-stabilized Pickering emulsions and to explore the non-covalent interaction between AZ and SA. Thus, providing new ideas for the development of novel, green, polysaccharide-protein composite food-grade Pickering emulsions for potential applications in fat-replacement foods and nutrient delivery.

## 2. Materials and Methods

### 2.1. Materials

Zein (from Zea mays L.Cat No.: 294946) was purchased from J&K. Sodium alginate (AR, 90%, S817374) was purchased from Macklin. Aminase bioassay test Kit (MAK310) was obtained from Sigma Aldrich Inc. (St. Louis, MO, USA). Soybean oil was purchased from the local market. Nile Red and Nile Blue A were purchased from Macklin Biochemical Co., Ltd. (Macklin, Shanghai, China). All other chemicals were of analytical grade.

### 2.2. Preparation and Characterization of ZS

#### 2.2.1. Preparation of AZ

The preparation of AZ was based on a previously reported method and slightly modified [28]. Zein was dissolved in ethanol solution (70%) containing NaOH (0.5 M) and reacted at 37 °C for 36 h. After that, the ethanol was removed under a vacuum. Then, 5 M HCl was added to the remaining solution to adjust its pH to 3.1, and the solution was precipitated overnight at 25 °C. The precipitate was separated and dissolved in water, and 2 M NaOH was added to an aqueous solution to adjust the final pH to 9.0 so that the peptide was completely deprotonated. Finally, the AZ powder was prepared by freeze-drying (Labconco FreeZone Vacuum Freeze Dryer, Labconco Corporation of America).

#### 2.2.2. Determination of the Degree of Deamidation (DD)

The AZ samples obtained with different deamidation reaction times were tested using the aminase bioassay test kit (MAK310). All the samples were diluted with 80% ethanol to a protein concentration of 4 mg/mL. Moreover, the probes were added quantitatively and left for 2 h. The absorbance at λ_max_ = 570 nm was recorded (Varioskan LUX Multi-function microplate reader, Thermo Fisher Scientific, Waltham, MA, USA). *DD* was calculated according to the following Formula (1):

(1) $$DD\%=\frac{I_{D}-I_{N}}{I_{S}-I_{N}}\times 100\%$$

where *I_D_* is the fluorescence intensity of the AZ dispersion; *I_N_* is the fluorescence intensity of the untreated fresh zein dispersion; *I_s_* is the fluorescence intensity of the zein dispersion treated with 3N sulfuric acid.

#### 2.2.3. Measurement of the Degree of Hydrolysis (DH)

*DH* was calculated according to the previous pH-Stat method, [29,30], and the formula was listed below (2):

(2) $$DH\%=\frac{h}{h_{tot}}=\frac{N_{b}\times B\times 100}{\alpha \times M_{p}\times h_{tot}}$$

where *N_b_* is the concentration of NaOH (M); *B* is the volume of NaOH consumed (mL); *M_p_* is the mass of hydrolyzed protein (g); *h_tot_* was the total number of peptide bonds per gram of protein substrate, and zein was 9.2 mmol·g^−1^. The *α* refers to the average dissociation degree of α-amino and pK at a specific pH value and temperature, and its value is 0.99.

#### 2.2.4. Fourier Transform Infrared Spectroscopy (FTIR) Measurements

The AZ obtained from the above reaction for 36 h was freeze-dried and the qualitative analysis of SA, zein, and AZ was performed by Fourier transform infrared spectrometer (Nicolet iN10, Thermo Fisher). Using the ATR attachment, the analysis was performed in the wavenumber range of 4000 to 400 cm^−1^ and 64 scans were performed with a resolution of 4 cm^−1^.

#### 2.2.5. Preparation and Characterization of ZS Particles

AZ was dissolved in ultrapure water to obtain a reserve solution with a final concentration of 20 mg·mL^−1^. In addition, SA was also dissolved in ultrapure water overnight to make it fully hydrated, and the final concentration of the polysaccharide solution was 5 mg·mL^−1^. The peptide solution and polysaccharide were mixed at the mass ratios of 1:1, 2:1, 3:1, 4:1, and 5:1, respectively, under the condition of continuous agitation at 25 °C for 0.5 h. The obtained dispersions were named Z1S1, Z2S1, Z3S1, Z4S1, and Z5S1, and stored at 4 °C.

#### 2.2.6. Particle Size and Zeta-Potential

According to the Mie scattering theory, the angle of the scattering light is inversely proportional to the diameter of the particle under the irradiation of the laser beam, and the intensity of the scattering light decreases logarithmically with the increase of the angle. The size of freshly prepared ZS particles was analyzed using a Mastersizer 2000 (Malvern Instruments Ltd., Worcestershire, UK), after dilution of the ZS dispersion. The zeta potential of the granular dispersions was tested using the Nano ZS + MPT-2 analyzer (Malvern Instruments Ltd., Worcestershire, UK).

#### 2.2.7. Surface Wettability

The surface wettability of the ZS particles was measured using an OCA 15EC water contact angle meter (Dataphysics Instruments GmbH, Stuttgart, Germany) equipped with a high-speed camera. The freeze-dried granule powder was pressed into tablets to obtain a diameter of 13 mm and a thickness of 2 mm. The tablet was immersed in the oil phase, and then the syringe was filled with ultrapure water, which was quickly added to the surface of the tablet using a high-precision syringe, and the image of the droplet was quickly captured by a high-speed camera. Each test was averaged three times [31].

#### 2.2.8. Field Emission Scanning Electron Microscope

The field emission scanning electron microscope (FESEM, S2018003810, Hitachi) was used to characterize the micromorphology of ZS particles with different proportions under the acceleration voltage of 2.0 kV. Before analysis, the freeze-dried samples were dispersed and dissolved, and a few drops were added to the tinfoil paper for natural drying or drying. The next step was gold spraying to avoid charging under the electron beam. Finally, the microscopic morphology was observed.

#### 2.2.9. Differential Scanning Calorimetry (DSC)

The thermal stability of the nanoparticles was characterized by DSC (Mettler-Toledo DSC, TA Instruments, Columbus, OH, USA). Approximately 3–5 mg of lyophilized samples were weighed into an aluminum crucible for analysis. The heating temperature was set from 25 to 250 °C, the heating rate was set to 10 °C/min, and the flow rate of nitrogen was set to 20 mL/min.

### 2.3. Preparation and Characterization of ZS-Stabilized Pickering Emulsion

#### 2.3.1. Preparation of ZS-Stabilized Pickering Emulsion

The ZS-stabilized Pickering emulsion was prepared under the condition of 2.5% total particle concentration. The volume fractions of the oil phase in the emulsions were 55%, 65%, and 75%, respectively. A high-speed homogenizer was used to homogenize three times at 19,000 rpm for 2 min each time. One part of the freshly prepared emulsion was stored at 4 °C, and its stability changes were observed by taking photos regularly, while the other part was used for subsequent related tests.

#### 2.3.2. Particle Size Distribution

The oil-droplet size of emulsions was analyzed for particle size distribution using Mastersizer 2000. Before testing, the emulsions were diluted at 25 °C. The refractive index of water and oil were 1.3330 and 1.4743, respectively, and the equilibrium time was 2 min.

#### 2.3.3. Kinetic Stability-Turbiscan Stability Analyzer

The kinetic stability of the emulsion was tested and analyzed using the Turbiscan stability analyzer (multiple light scatterers) (Formulation Turbiscan). The stability of the emulsion was investigated by measuring the transmittance and backscatter of pulsed near-infrared light (*λ* = 880 nm). About 25 mL of emulsion samples were placed in special test bottles and placed in the Turbiscan test chamber. The detector scanned each sample bottle vertically at 25 °C for a total of 10 h and automatically recorded the results. The Turbiscan stability index (TSI) is calculated by Turbisoft Lab software to reflect the overall stability of the emulsion, and it can be calculated by the following Formulas (3) and (4) [32]:

(3) $$BS=1/\sqrt{\lambda^{*}},\lambda^{*}\left(\varphi ,d\right)=\frac{2d}{3\varphi \left(1-g\right)Qs}$$

(4) $$TSI={\left(\frac{1}{n-1}{\sum_{i=1}^{n}\left(x_{i}-x_{BS}\right)}^{2}\right)}^{1/2}$$

where λ* is the photon transport mean free path in the analyzed dispersion; *φ* is the particle volume fraction; *d* is the mean diameter of the particles; *g* and *Qs* are the optical parameters from Mie’s theory; *x_i_* is the average backscattering for each minute of the test; *x_BS_* is the average of *x_i_* and *n* is the number of scans.

#### 2.3.4. Micromorphology

The micromorphology of the Pickering emulsion stabilized by ZS particles was observed by a confocal laser scanning microscopy (CLSM, LEICA SP, LEICA Microsystems Inc., Heidelberg, Baden-Wurttemberg, Germany). First, Nile Red (0.1%) and Nile Blue (0.1%) dyes were configured and mixed after both were dissolved. The emulsion was then dyed. In this experiment, 1 mL of emulsion and 20 μL of mixed dye were mixed and then dropped onto a clean glass slide, the coverslip was covered, and the slide was sealed if necessary. The excitation wavelengths were 488 nm and 638 nm when observed with a 63× objective lens.

#### 2.3.5. Rheological Property

The rheological properties of the freshly prepared emulsion were analyzed using an Anton Paar rheometer (MCR92). For steady-state shear measurements, the viscosity was measured at shear rates from 0.1–100 s^−1^. The angular frequency was 0.1–100 rad·s^−1^ carries out a frequency scan test. The strain value of the emulsion was determined by analyzing it in the linear viscoelastic region. Finally, the operating values of modulus of elasticity (*G*′) and loss modulus (*G*″) as a function of angular frequency are obtained.

### 2.4. Statistical Analysis

Each test was repeated three times, and all data were expressed as mean ± standard deviation (SD). Statistical analysis was performed with SPSS software (IBM, Chicago, IL, USA). Duncan′s multiple range tests were used to analyze the variance (ANOVA) of the obtained data. *p* ≤ 0.05.

## 3. Results and Discussion

### 3.1. Characterization

#### 3.1.1. Determination of the DD and DH of AZ

**Figure 1:**
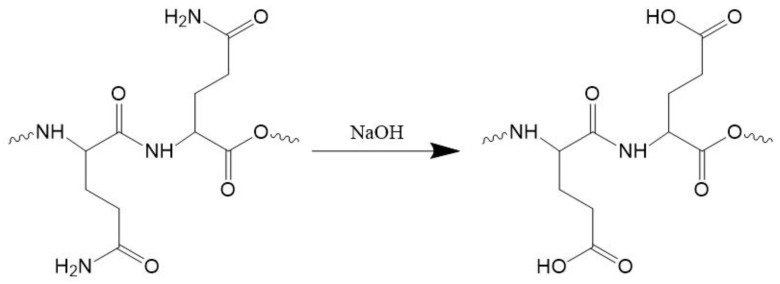
Schematic diagram of deamidation.

Deamidation of zein through alkaline heating could improve its molecular flexibility and confer a strong structural inversion ability to zein [33,34,35]. Therefore, zein was first modified by restricted alkali deamidation with the reaction equation shown in Figure 1. The *DD* of AZ was calculated to be 12.74% and the *DH* to be 6.58%, indicating that the alkali pretreatment did not cause significant main chain breaks in zein. This result may be attributed to the mild method of alkali treatment in this study, which mainly caused glutamine deamidation on the side chain of the protein, while no substantial hydrolysis of the peptide bonds in the main chain occurred. Previous studies have shown that low *DH* (<15%) can promote the improvement of emulsion stability [28,36,37]. Peptides obtained by excessive hydrolysis of zein proteins tend to destabilize the emulsion, probably due to an inappropriate hydrophobic-hydrophilic balance [38]. Accordingly, the DH of AZ was controlled at a low level in this study.

#### 3.1.2. FTIR Analysis

**Figure 2:**
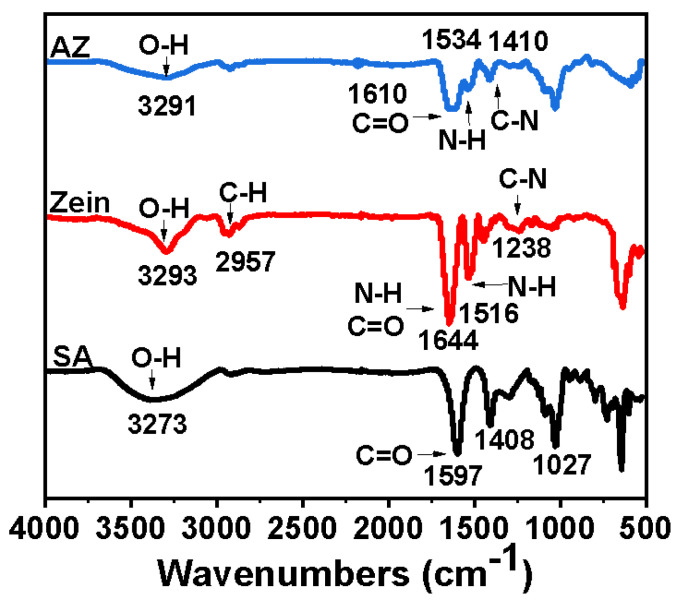
FTIR spectra of alkali-treated zein (AZ), zein, and sodium alginate (SA).

FTIR spectroscopy provides information on the molecular structure and chemical bonding of actual materials through radiation absorption caused by molecular vibration and can be used for the chemical analysis of protein–polysaccharide. The FTIR spectra of SA, zein, and AZ are shown in Figure 2. Characteristic peaks of zein and SA appeared at 3293 and 3273 cm^−1^, respectively, which were caused by tensile vibrations of O-H [24,26]. The characteristic peak of zein was observed at 2957 cm^−1^, which was related to the C-H stretching vibration of the alkyl groups. In addition, the characteristic peak of zein at 1644 cm^−1^ was attributed to the N-H bending vibration peak in the primary amide II band, which overlapped with the C=O tensile vibration peak. The N-H bending vibration peak of the secondary amide appears at 1516 cm^−1^, and the C-N stretching vibration peak of amide III appears at 1238 cm^−1^ [39]. After alkali heat treatment of zein, the AZ obtained mainly appeared characteristic peak at 1610 cm^−1^, which was the stretching vibration peak of C=O. At 1534 and 1410 cm^−1^, N-H bending vibration peaks and C-N stretching vibration peaks of the amide II band and amide III band, respectively, were observed. Compared with the natural zein, after alkali heat treatment, the position of the primary amide peak in the amide II band became wider and the intensity decreased significantly, and the O-H at the position of 2700–3700 cm^−1^ became wider, indicating that alkali heat treatment was mainly to remove the free primary amide on the surface of zein, and the intramolecular secondary amide hydrolysis was less. To some extent, this marks the success of AZ preparation.

#### 3.1.3. Particle Size and Zeta-Potential

**Figure 3:**
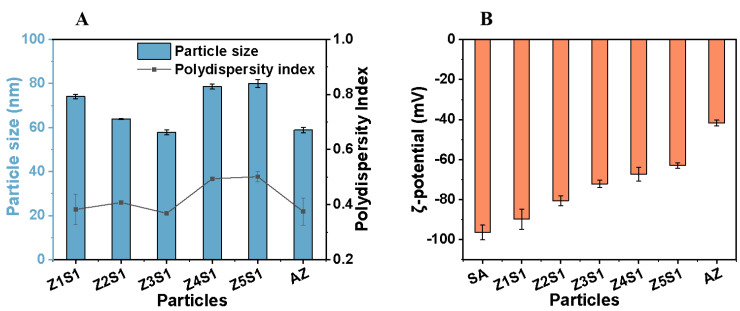
(**A**) AZ and ZS particle size, Polydispersity index (**B**) Zeta potential of ZS particles with different ratios.

The particle sizes, PDI, and zeta-potentials of AZ and ZS with different ratios are shown in Figure 3A. Untreated zein has a particle size of up to 172 nm. After deamidation, the particle size of AZ decreased to 58.82 nm, and the PDI of AZ dispersion was 0.37, indicating that alkali treatment and anti-solvent preparation could significantly shrink the particle size of zein and enhance its dispersibility in an aqueous solution. The particle size of ZS complexes with different proportions was not more than 80 nm, and the PDI of ZS complexes was between 0.30~0.50, suggesting that the surface modification of AZ by SA has little effect on the particle size and dispersion. With the increase of AZ content, the particle size and PDI of ZS complexes first decreased and then increased. Among the five ZS complexes, Z3S1 showed the smallest particle size and PDI, which were 57.72 nm and 0.37, respectively. This may be due to the fact that as the proportion of polypeptide molecules in the system increase, the carboxyl negative ions of SA are simultaneously hydrated, the polypeptide molecules expand in polar solvents, and the volume increases. SA and AZ form liquid and compact spherical aggregates, leading to an increase in particle size [35,40].

The zeta potential is a crucial factor in maintaining the stability of the colloidal system. The stability of particles in solution is mainly based on the following two mechanisms: electrostatic repulsion and steric hindrance effect. When the zeta potential of a stable system is very low, a steric effect may exist. However, at high potential, electrostatic repulsion exists and the sample remains stable. Under low potential and zero potential conditions, flocculation, agglomeration, and precipitation of the ZS particle dispersion system can easily occur. As shown in Figure 3B, at a low proportion of AZ, the absolute potential of the system is large and the whole system has a negative potential, indicating that electrostatic repulsion is dominant in the whole system at this time. However, as the AZ/SA ratio increases, the potential of the ZS particles gradually decreases. This is because both SA and AZ contain a large number of carboxyl groups that can hydrate with water. The solution system formed by SA and water has a high viscosity, which can wrap a certain amount of AZ particles. However, as the AZ content in the system increases, the uncoated AZ particles are dispersed in the solvent and may agglomerate and precipitate, leading to the instability of the system, the gradual decrease of the potential value, and the reduction of the electrostatic repulsion of the system. In addition, the system tends to aggregate, the attractive force exceeds the repulsive force and gradually dominates, and the anti-aggregation ability of the system is weakened, which is bad for the stability of the emulsions.

#### 3.1.4. Surface Wettability

Many factors influence the surface wettability of proteins. For example, zein is an amphiphilic protein, but its surface wettability is usually affected by the processing conditions and preparation methods. The samples obtained by the evaporation-induced self-assembly method exhibit moderate wettability because the molecules form self-assembled and ordered layered structures, which are more suitable for the development of biofilms in the food field. However, for Pickering emulsion, a complete particle structure is required for adsorption at the oil–water interface, so the sample preparation method and wettability are different. In this study, to better preserve the original structure of zein, zein, and AZ particles were prepared by freeze-drying throughout the experiment and their surface hydrophobicity was characterized.

**Figure 4:**
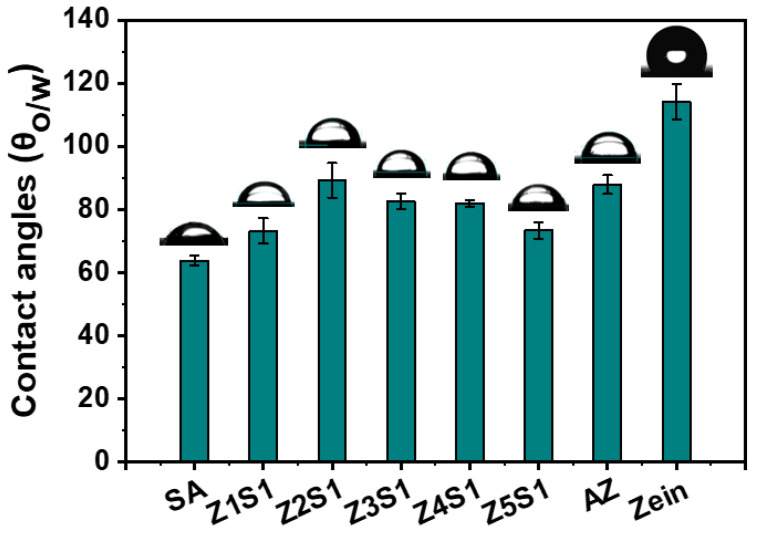
The surface wettability of ZS particles with different ratios.

The surface wettability of the ZS particles was measured by examining the three-phase contact angle (*θ*_o/w_), as shown in Figure 4. It is reported that the proximity of the *θ*_o/w_ to 90° is a key factor for the presence of kinetically stable Pickering emulsion [10]. The results show that the *θ*_o/w_ of zein is 114.0°, indicating the relative hydrophobicity and poor wettability of its particles. The *θ*_o/w_ of AZ particles was reduced by about 87.83° compared to that of natural zein, indicating that the hydrophilicity of zein modified by alkali treatment was improved. The *θ*_o/w_ of Z1S1, Z4S1, and Z5S1 were 73.13°, 81.77° and 73.27°, respectively, showing the relative hydrophilicity of their particles. The *θ*_o/w_ of Z2S1 and Z3S1 were 89.13° and 82.53°, respectively, suggesting the best amphiphilic properties among the ZS complexes.

#### 3.1.5. Morphology

**Figure 5:**
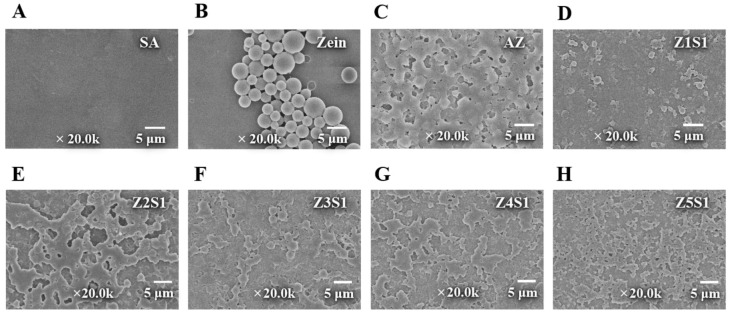
FESEM of SA, zein, AZ, and ZS particles with different ratios.

Figure 5 shows the microscopic morphology of the ZS particles obtained by FESEM. It could be seen that after deamidation, the zein molecules were partially swollen and digested, and the spherical structure collapsed. In the complex system, the carboxyl anions hydrated simultaneously hydrated, and SA and AZ formed liquid, compact spherical condensates. At the same time, some aggregate and linkage of the ZS particles were observed, which may be due to the bridging effect of SA adsorption on the nanoparticle surface [41,42].

#### 3.1.6. Differential Scanning Calorimetry (DSC)

**Figure 6:**
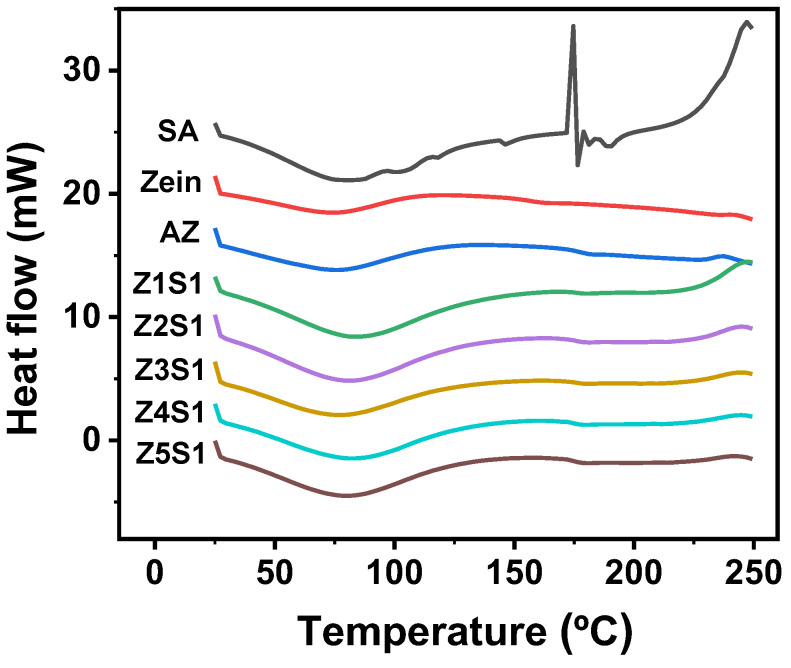
Differential scanning calorimetry (DSC) curves of SA, zein, AZ, and ZS particles with different ratios.

As shown in Figure 6, the characteristic endothermic peaks of zein and AZ appear at 80.88 °C and 80.86 °C, respectively, which are caused by energy absorption during the evaporation of bound water in the sample. The peak of zein is near 110 °C, indicating the onset of thermal denaturation. However, the thermal denaturation temperature of AZ obtained by alkali treatment was 125 °C. The results showed that deamidation improved the thermal stability of zein and increased the thermal denaturation temperature. In addition, SA showed an obvious endothermic peak at 90.15 °C and complete melting at 176 °C. For the ZS complexes, with the increase of SA content, the endothermic peak of ZS gradually shifted towards higher temperatures. The results indicated that the addition of SA enhanced the electrostatic interactions between the different components of the complex, resulting in the higher temperature of the endothermic peak, thus improving the thermal stability of the proteins.

### 3.2. Characterization of the Pickering Emulsion

#### 3.2.1. Droplet Size and Storage Stability

**Figure 7:**
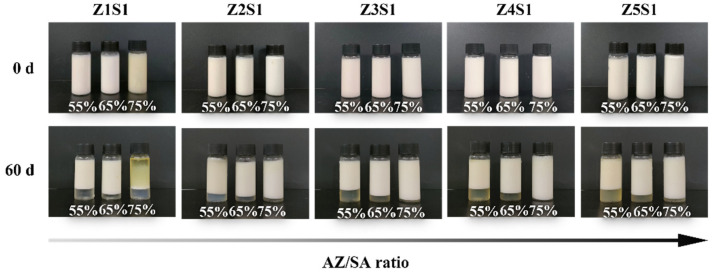
The visual appearance of Pickering emulsions stabilized by ZS particles with different oil part fractions at 0 d and 60 d.

**Figure 8:**
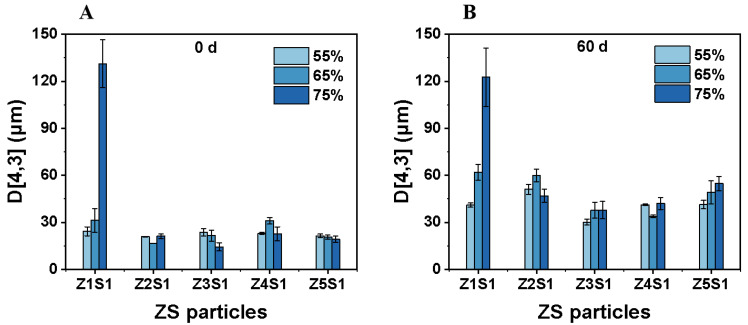
The mean volume diameter of the Pickering emulsions stabilized by ZS particles with different oil part fractions at 0 d (**A**) and 60 d (**B**).

Macroscopic photos of Pickering emulsions stabilized by ZS with an oil–water ratio between 55% and 75% at 0 d and 60 d are shown in Figure 7. Moreover, their oil-droplet size distribution is shown in Figure 8. The result showed that no phase separation occurred in the freshly prepared Pickering emulsion stabilized by ZS particles. However, after 60 d of storage, most of the emulsions stabilized by ZS particles remained stable, with a small amount of clarification layer appearing at the bottom, but no oil phase was seen in the upper layer of the emulsions, except for the Pickering emulsions stabilized by Z1S1 particles with 75% oil phase volume fraction. The clarification layer appeared in the emulsions with 55% and 65% oil phase volume fractions with completely different ZS proportions, and the height of the clarification layer increased slightly with the increasing AZ/SA ratio. The Z2S1 particle-stabilized Pickering emulsions with 75% oil phase fraction maintained a uniform appearance and no phase separation appeared after 60 d. For Z1S1-stabilized emulsions, the stability of the emulsions gradually decreased with the increase of the oil phase volume, and the oil-droplets size increased two times after 60 d, as well as the presence of oil phase precipitation in the emulsions with 75% oil phase fraction. This may be due to the excessive potential on the surface of Z1S1 particles, which instead leads to their easy destabilization and agglomeration so that the ZS particles at the oil–water interface are not able to completely cover the oil droplets [43]. On the contrary, the oil-droplet size of most of the other emulsions decreases with the increase in the volume fraction of the oil phase, indicating that the storage stability of emulsions with higher AZ/SA ratios could be improved by increasing the volume fraction of the oil phase. This may be due to an increase in the viscosity of the emulsion (Section 3.2.4), which slows down the gravitational separation according to Stokes’ law. It has also been shown that the oil fraction plays an important role in emulsion flocculation [44]. Due to the coalescence of some oil droplets during storage, the oil-droplet size of the emulsion increases to some extent after 60 d. The Z3S1-stabilized emulsions showed the smallest oil-droplet size both before and after 60 days of storage, so they were selected for further kinetic stability analysis.

#### 3.2.2. Kinetic Stability Analysis

**Figure 9:**
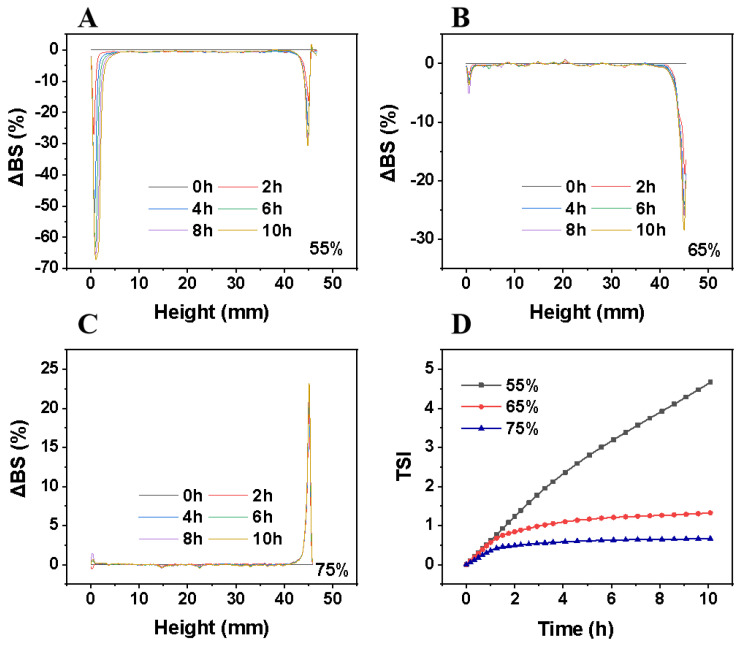
The backscattered light (ΔBS) of emulsions stabilized by Z3S1 with different oil phase volume fractions, φ = 55% (**A**), φ = 65% (**B**), φ = 75% (**C**). TSI value of emulsions stabilized by Z3S1 with different oil fractions (**D**).

The backscattered light (ΔBS) versus measurement tank height and time for the Z3S1-stabilized emulsions are shown in Figure 9A–C. With the oil phase fraction of 55%, a small amount of particle sedimentation occurs in the emulsion and the concentration at the bottom of the sample bottle increases. As the volume fraction of the oil phase increases, there is no floating or settling of particles at the bottom and top of the emulsion bottle, which remains stable. According to Stokes’ law, the larger the radius of the emulsion droplet, the larger the gain speed, which is able to accelerate the upward motion of the oil droplet, and therefore, the stability of the emulsion decreases, and conversely, the stability of the emulsion increases [45,46].

As shown in Figure 9D, the TSI value of the emulsions stabilized by Z3S1 particles decreases with the increase of the volume fraction of the oil phase, which means that the stability of the emulsion is improved. This may be due to interionic hydration, because deamidation converts the amide group on the zein molecule to a carboxyl group, and SA contains carboxyl negative ions, that can hydrate with water. When two emulsion droplets are close to each other, the interaction between the polar groups and the nearby water is destroyed, resulting in a repulsive interaction that prevents the particles from aggregating and settling, and maintains the stability of the emulsion [47,48]. In general, the TSI values of the emulsions were negatively correlated with the volume fraction of the oil phase, and the final emulsions prepared were kinetically stable.

#### 3.2.3. Micromorphology

**Figure 10:**
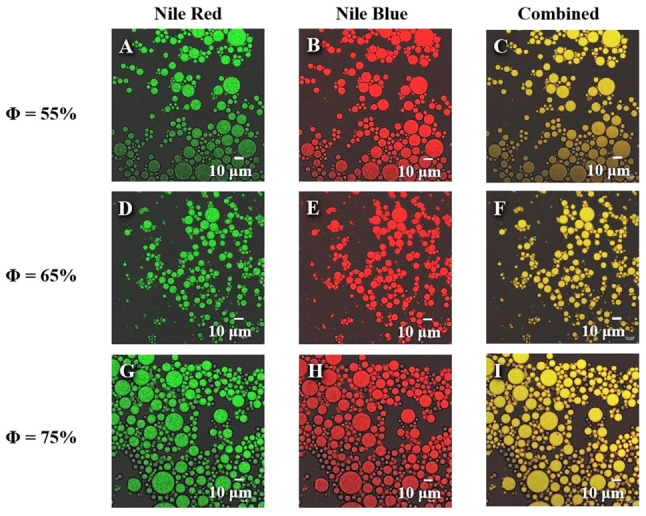
CLSM images of Pickering emulsions stabilized by Z3S1 with different oil fractions.

The microstructure of Pickering emulsions provides a deeper insight into the interfacial properties of the emulsion. Therefore, CLSM was used to observe the interfacial microstructure of the emulsions (Figure 10). The oil droplets and protein colloidal particles in the emulsions were stained with a mixture of Nile Red and Nile Blue dyes, respectively. The green and red fluorescence indicates the presence of the oil phase and colloidal particles. As can be seen in the figures, the ZS particles form a coating around the oil droplets, causing the steric effect. It is also possible that the negative charge of the ZS coating causes electrostatic repulsion to stabilize the emulsions [49]. Furthermore, the emulsion droplets decreased slightly with the increase of the dispersed phase volume fraction, which is in step with the results of the particle size characterization. Moreover, CLSM also observed that the droplets remained independent, and relatively few small oil droplets coalesced into large oil droplets, which is consistent with the macroscopic observation [50].

#### 3.2.4. Rheological Property

**Figure 11:**
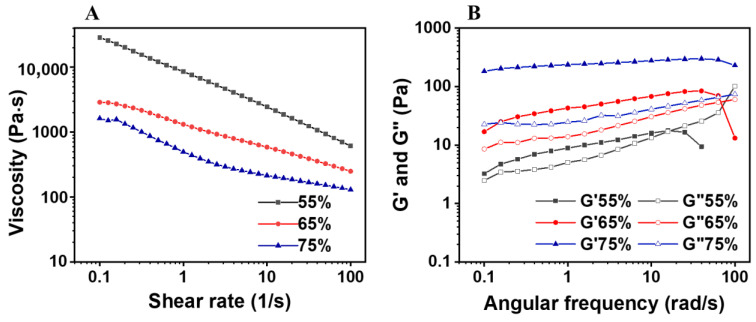
The relationship between viscosity and shear rate for Pickering emulsions with different oil fractions (55%, 65%, and 75%) (**A**). G′ and G″ as a function of frequency for Pickering emulsions with different oil fractions (55%, 65%, and 75%) (**B**).

Viscosity is the internal friction that occurs when all the components of the fluid are forced to slide against each other. As shown in Figure 11A, the viscosity of the emulsion was first characterized. With the shear rate of 0.1–100 s^−1^, the viscosity of the emulsion gradually decreased, indicating that the emulsion had shear thinning behavior and belonged to a non-Newtonian fluid. The apparent viscosity of the emulsion increased significantly with the decrease of the oil phase volume fraction [21].

The dynamic oscillations of the emulsion were then measured in the range of the linear viscoelastic region, with frequencies ranging from 0.1–100 rad/s. It can be seen from Figure 11B that for the emulsions stabilized by Z3S1 particles with an oil phase fraction of 75%, G′ was higher than G″, indicating that the energy storage modulus of the emulsion was dominant and the overall solid-like behavior was presented. The emulsion mainly underwent elastic deformation (G′ > G″). Moreover, when the volume fraction of the oil phase is 55% and 65%, the phase reversal occurs at different angular frequencies. As the volume fraction of the oil phase increases, the transition points of the energy storage modulus and loss modulus move to low angular frequencies. This may be due to the fact that this experiment was performed under alkaline conditions, as at lower pH the carboxyl group is completely protonated, and SA forms flocs. However, under alkaline conditions, G′ and G″ have a little dependence over the frequency scanning range of all Pickering emulsions, and the oil droplets are encapsulated in the network formed by the ZS particles, and their migration is limited to some extent. This demonstrates that the non-covalent physical interactions are the main driving force for the formation of gel-like network structures in emulsions [51,52].

## 4. Conclusions

In this work, zein was treated by alkaline deamidation, and the obtained AZ and SA were mixed in different proportions as the emulsifier to stabilize the emulsion. The results showed that the DD and DH of AZ obtained by deamidation for 36 h were 12.74% and 6.58%, respectively. FTIR showed that the deamidation reaction mainly occurred at glutamine in the side chain of zein. The addition of SA significantly increased the charge density of the AZ surface. The *θ*_o/w_ values of Z2S1 and Z3S1 particles are 89.13° and 82.53°, respectively, indicating the best wettability at the interface. Emulsions with oil phase volume fractions of 55%, 65%, and 75% were prepared, and their stability was characterized and analyzed. It was observed that Z3S1 particles formed a coating at the oil–water interface, resulting in the smallest oil-droplet size and stable appearance of the emulsion before and after 60 days of storage. With the decrease of the volume fraction of the oil phase, the viscosity of the emulsion gradually increases and exhibits an overall solid behavior. In conclusion, the strong electrostatic repulsion in the AZ–SA system significantly reduces the possibility of colloidal particle aggregation and improves interfacial stability. Hydration and electrostatic repulsion are considered to be the main driving forces for the stability of ZS emulsions.

## Data Availability

The data presented in this study are available through email upon request to the corresponding author.

## Sample Availability

Samples of the compounds are not available from the authors.
