# Correction: Kuang et al. Investigation and Characterization of Pickering Emulsion Stabilized by Alkali-Treated Zein (AZ)/Sodium Alginate (SA) Composite Particles. *Materials* 2023, *16*, 3164

**DOI:** 10.3390/ma18112511

**Published:** 2025-05-27

**Authors:** Ying Kuang, Qinjian Xiao, Yichen Yang, Menglong Liu, Xiaosa Wang, Pengpeng Deng, Kao Wu, Yi Liu, Bo Peng, Fatang Jiang, Cao Li

**Affiliations:** 1 Cooperative Innovation Center of Industrial Fermentation (Ministry of Education & Hubei Province), Hubei Key Laboratory of Industry Microbiology, National “111” Center for Cellular Regulation and Molecular Pharmaceutics, Key Laboratory of Fermentation Engineering (Ministry of Education), Hubei University of Technology, Wuhan 430068, China; 2 Department of Architecture and Built Environment, Faculty of Engineering, University of Nottingham, Nottingham NG7 2RD, UK; 3 College of Health Science and Engineering, Hubei University, Wuhan 430062, China

In the original publication [1], there was a mistake in Figure 7 as published. The image of the Z4S1-stabilized emulsions at day 0 was incorrect. The corrected Figure 7 appears below. The authors state that the scientific conclusions are unaffected. This correction was approved by the Academic Editor. The original publication has also been updated.

## Figures and Tables

**Figure 7:**
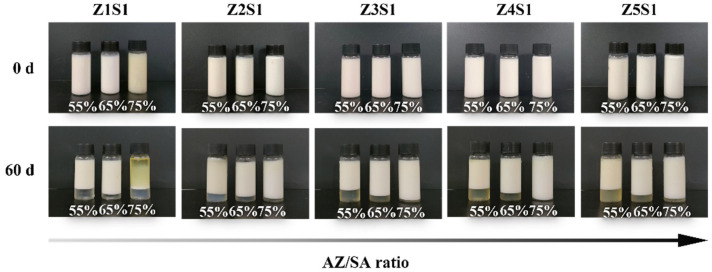
The visual appearance of Pickering emulsions stabilized by ZS particles with different oil part fractions at 0 d and 60 d.
